# Concerning the Etiology of Dental Caries

**Published:** 1914-09-15

**Authors:** C. F. Bodecker

**Affiliations:** Berlin, Germany


### CONCERNING THE ETIOLOGY OF DENTAL CARIES.

BY C. F. BODECKER, D. D. S., BERLIN, GERMANY.

Dr. J. Sim Wallace of London published a book, now in its second edition, on "The Prevention of Dental Caries," a subject of undoubtedly universal interest.

"Pappy" Food as a Causative Factor of Caries.—The principal thought set forth is that caries is caused by the "pappy" consistence of the food now eaten by the civilized races of the world. He claims that if we would eat food of a more fibrous nature, or at least finish our meals with such food, the teeth would be cleansed of adhering particles, and decay would not take place. He advises that raw fruit be eaten after every meal, the apple being the best, as it requires thorough mastication.

About this there is no doubt, for it has been the belief of most dentists that through lack of use teeth deteriorate, and therefore decay more easily. If man would eat his food in an uncooked state, as was intended by nature, the thorough mastication needed would automatically keep the teeth spotlessly clean, and he would not have the slightest need of a tooth-brush. But our digestion would now protest against raw food, so that we have to resort to artificial cleansing and massage of teeth and gums. We have all heard the war-cry of Dr. Cunningham, "Clean teeth do not decay," and although this is not true in all cases, we had best let the public think so—for if the masses were given the idea that there is some doubt about this claim, they might neglect the tooth-brush as a useless expense and inconvenience, and their teeth would undoubtedly suffer.

W. D. Miller's Work on Caries Unjustly Discredited.—Dr. Wallace expresses in his book the opinion that W. D. Miller did not discover the cause of caries, an opinion which seems to be shared by a number of our English colleagues.

I can do no better than repeat two quotations which Dr. Wallace cites to prove that the cause of caries was unknown up to the present:

There seemed to be a general belief, at any rate among those practicing dentistry, that the cause of caries had been found, and the fact seemed to be lost sight of that all that Dr. Miller had done was to demonstrate the phenomena of caries, and although his researches had shown the channels in which further investigation should be pursued, the actual cause of caries was still unknown, and is to the present day unknown. (Leading article, published in the *Lancet*, October 21, 1905.)

The second quotation is taken from Stanley P. Mummery, "Heredity and Dental Diseases," proceedings of the Royal Society of Medicine, 1908, p. 108:

It is frequently stated that the true cause of dental caries has yet to be discovered, and although we know the pathology of the disease through the investigations of Dr. Miller, the etiology is a very different matter.

Another part of the quotation from the *Lancet* reads as follows:

If we wish to deal with dental disease, we must first of all possess a correct knowledge of its cause. Do we possess that knowledge? It is now more than fifteen years since Dr. W. D. Miller's excellent work on the "Micro-organisms of the Mouth" appeared, in which he clearly demonstrated that caries of the teeth was due to the abstraction of the lime salts by acid, followed by the peptonizing action of bacteria, the acids being formed from the fermentation of carbohydrate food.

Dr. Wallace's Alleged Discovery Regarding the Etiology of Caries.—On the next page (page 27) Dr. Wallace gives us his version, printed in italics, of the true cause of caries:

The cause of caries is the prolonged retention or stagnation of fermentable carbohydrates in more or less immediate contact with the teeth, and undisturbed by the free access of saliva.

Can anyone read the above passages and still claim that W. D. Miller did not discover the cause of caries?—for there is no difference between what Miller is quoted as having accomplished and Wallace's definition. The latter is worded more simply, while Miller used scientific terms. Dr. Wallace has confused the terms *exciting* and *predisposing* causes of caries, which come under the head of etiology. If we take pneumonia, for example, the predisposing causes are alcoholism, exposure, typhoid, gastro-intestinal diseases, etc., whereas the exciting cause is the diplococcus pneumoniae. Yet we find this germ in the throat of almost every person, even though he be healthy, which proves that unless the predisposing cause is present, the diplococcus pneumoniae will do no harm. This is analogous to the phenomena of caries. In this disease the exciting cause is the formation of acid through the action of bacteria upon carbohydrate food, and there can be no doubt that W. D. Miller discovered this fact. But very little attention has been given to the predisposing causes of caries. Irregularities, causing food to lodge easily between the teeth, may be a predisposing cause, but the principal one is to be sought in the tooth tissues themselves. To this question I will return later.

THE PROBLEM OF SUSCEPTIBILITY AND IMMUNITY.—As the writer in the *Lancet* did not accept Miller's theory as to the cause of caries, it is to be doubted whether he will consider Dr. Wallace's ideas as being nearer the truth. For the two are the same. If previous to Dr. Wallace's book it had been unknown that, usually, ill-cleaned teeth decay easily, why have strenuous attempts been made to remove all traces of food from the teeth by means of a tooth-brush and floss silk? Every student for the last decade has heard throughout his college training that wherever food lodges for any length of time, decay will take place. This is true from the theoretical aspect, but regarded from the clinical point of view—does decay *always* take place where food lodges? It does not! For this reason, the writer in the *Lancet* says that the actual cause of caries is still unknown. (Presumably he means the predisposing cause.) Fermentable carbohydrates often cause caries, but every practitioner has seen extensive approximal caries on one molar or bicuspid, whereas the adjoining tooth was still in a healthy condition. There must still be some factor missing to explain this immunity.

This brings us to susceptibility and immunity to caries. We will first consider this in the general sense in cases where one mouth seems to be immune, while another is totally wrecked by caries. This difference may be explained in part, but in part only, by the difference in diet as mentioned in Dr. Wallace's book. There is no doubt that some owe their carious teeth to filth, whereas there are other mouths, also excessively filthy, that are showing no trace of caries. Dr. Wallace accounts for this phenomenon through an incorrect diet or only an incorrect sequence of food. The patient should finish each and every meal with detergent foodstuffs, such as apples. If the only cause of caries were the lodging of foodstuffs on the teeth, there is no doubt that, by the correct choice of diet, carious teeth would become unknown. Dr. Wallace remarks:

Susceptibility and immunity, in regard to dental caries, is, if not a misconception altogether, at least an almost negligible factor, which, at the present day throws no light whatever on the means at our disposal for preventing the disease.

Susceptibility has become with many a negligible factor, because it has been difficult to find radical histological differences in the teeth of patients susceptible and those immune to caries. Yet it is only by the study of the predisposing causes of caries that means will be found for combating this scourge.

### The Role of the Enamel: "Hard" and "Soft" Teeth.

—In connection with this question, the histology of the enamel of the teeth plays the greatest role, for after that structure has succumbed to caries, the dentin offers but little resistance. Teeth were at one time divided into "hard" and "soft" teeth—that is, the hard ones were those immune to caries, while the soft ones were regarded as prone to decay. This classification was attacked and apparently refuted by innumerable histologists, so that no one dares to mention it. Yet almost every practitioner has the feeling that many of the teeth of patients prone to acute caries are decidedly softer than others. Black states that in the excavation of a carious cavity the enamel is found to be also carious, and therefore softened through partial decalcification. That is true, but when we extend the cavity margins, according to the teachings of Black, beyond the contact point to prevent secondary caries, we have an excellent opportunity for testing the hardness of the healthy enamel. And there we still find hard or soft enamel, as the case may be. A second point in favor of the theory that hard and soft teeth exist is the possibility of soft teeth becoming hard again after the body has recovered from some constitutional ailment. This fact is denied by most histologists, yet every practitioner has seen white spots in the enamel, such as are sometimes found in the teeth of pregnant women, disappear. A third point in favor of the above theory is the fact that orthodontists massage white spots in the enamel that are sometimes caused by regulating appliances. If these white spots had no possibility of recovering, what good could be expected from massage? If it were to remove the decalcified enamel, why not excavate thoroughly and place a filling? Because the vitality of the enamel has been doubted is the reason why the histologists have not admitted that circulation exists. That is the crucial point, for without circulation the enamel could not change, except for the worse through caries. It has been my good fortune, after eleven years of work, to be the first to prove the presence of organic matter in the enamel. And it is this organic matter through which slight circulation takes place.

Immunity Due to Structure of the Enamel.—I would like to discuss one point more, and that is the local immunity to caries of a tooth the neighbor of which shows a large cavity. This immunity of the intact tooth cannot be explained by claiming that the lactic acid formed from food deposits could not act upon it. The food is wedged between the two teeth, and the acid-forming bacteria attack it from all sides; thus both teeth are under the influence of the acid, and both should decay with equal rapidity. The fact that they do not, irrefutably proves that some teeth are more resistant to decay than others. Some authors ascribe this immunity to the action of the saliva, but in these cases of local immunity, which we see most every day, not even the saliva can have this beneficial effect. *Nothing but a difference in the structure of the enamel can explain immunity to caries.*

Dr. Wallace is so optimistic as to the beneficial effect upon the teeth from the use of detergent foods after each meal, that he says (page 2) we shall hear of—

the decimation of the ranks of the dentists, which will follow when once the eyes of the public are opened to this scourge and its possibilities of prevention.

There can be no doubt that Dr. Wallace's book has filled a long-felt want in dental literature, for it emphasizes the fact that most people do not give proper attention to the care of their teeth, and it tells them that, on making the correct choice of foods, the teeth will be cleansed through mastication.—*Dental Cosmos.*

